# Laboratory survey of drug-resistant Streptococcus pneumoniae in New York City, 1993-1995.

**DOI:** 10.3201/eid0401.980116

**Published:** 1998

**Authors:** R Heffernan, K Henning, A Labowitz, A Hjelte, M Layton

**Affiliations:** New York City Department of Health, New York, USA.

## Abstract

Wide geographic variation in the prevalence of drug-resistant Streptococcus pneumoniae demonstrates the importance of tracking antimicrobial resistance locally. This survey of hospital microbiology laboratories in New York City found that penicillin resistance (MIC > or = 2.0 micrograms/ml) increased from 1.5% of S. pneumoniae isolates in 1993 to 6.3% in 1995 and that in 1995, one-third of isolates nonsusceptible to penicillin (MIC > or = 0.1 microgram/ml) were also nonsusceptible to an extended-spectrum cephalosporin (MIC > or = 1 microgram/ml).


### Laboratory Survey of Drug-Resistant *Streptococcus pneumoniae* in New York City, 1993–1995

Wide geographic variation in the prevalence of drug-resistant *Streptococcus pneumoniae* demonstrates the importance of tracking antimicrobial resistance locally. This survey of hospital microbiology laboratories in New York City found that penicillin resistance (MIC ≥ 2.0 µg/ml) increased from 1.5% of *S. pneumoniae* isolates in 1993 to 6.3% in 1995 and that in 1995, one-third of isolates nonsusceptible to penicillin (MIC ≥ 0.1 µg/ml) were also nonsusceptible to an extended-spectrum cephalosporin (MIC ≥ 1 µg/ml).

The emergence of drug-resistant *Streptococcus pneumoniae* underscores the need for timely, local, population-based surveillance of antimicrobial resistance. The prevalence of resistance in U.S. communities varies widely, with 2% to 53% of *S. pneumoniae* isolates found to have reduced susceptibility to penicillin (1-4). The Centers for Disease Control and Prevention recommends that empiric antibiotic therapy for pneumococcal infections be based upon local susceptibility patterns (2,5). However, few communities track drug-resistant *S. pneumoniae*.

### The Survey

To estimate the prevalence of drug-resistant *S. pneumoniae* in New York City, we surveyed hospital-based clinical microbiology laboratories from 1993 to 1995. A standardized questionnaire was mailed annually to each laboratory, and those that did not respond were contacted by telephone or were visited. To evaluate compliance with *S. pneumoniae* penicillin susceptibility testing guidelines established by the National Committee for Clinical Laboratory Standards (NCCLS) (6), we asked about criteria for selecting specimens and techniques for oxacillin disk diffusion screening and determination of penicillin MICs.

To determine the prevalence of penicillin resistance, we asked for the number of *S. pneumoniae* isolates identified during the year, the number tested for susceptibility to penicillin, and the number found to be possibly resistant by the oxacillin disk diffusion test and penicillin-intermediate or -resistant by MIC testing. We also asked that information be provided separately for isolates from normally sterile sites (e.g., blood, cerebrospinal fluid) and from nonsterile sites (e.g., sputum, nasopharyngeal swab). In 1995, we added questions regarding the MIC test results for extended-spectrum cephalosporins (ESCs), including the number of penicillin-nonsusceptible isolates that were also nonsusceptible to an ESC. No individual patient information was obtained. A report summarizing the results of the survey and describing NCCLS guidelines was mailed annually to microbiology laboratories, hospital infection control departments, and local infectious disease physicians and pediatricians.

### Analysis

Of 67 hospital-based clinical microbiology laboratories in New York City, 100% completed the survey in 1993, 98% in 1994, and 100% in 1995. Overall, more than 5,000 *S. pneumoniae* isolates were reported annually.

Data were analyzed by using EpiInfo Version 6.0 (CDC, Atlanta, GA, USA). Drug-susceptibility results are presented for laboratories that conformed with NCCLS guidelines and provided complete data on all *S. pneumoniae* isolates identified (Table 1).

### Susceptibility Criteria

The NCCLS recommends routine screening by the oxacillin disk diffusion test of clinically important *S. pneumoniae* isolates for susceptibility to penicillin. Isolates with a zone size ≤ 19 mm, or any isolate from the blood or cerebrospinal fluid, should be tested with an approved MIC method such as broth dilution or antibiotic gradient strips (e.g., E-test). Isolates whose penicillin MICs are either intermediate (MIC ≥ 0.1 and ≤ 1 µg/ml) or resistant (MIC ≥ 2 µg/ml) should also have MICs determined for susceptibility to an

Table 1. Penicillin resistance among *Streptococcus pneumoniae* isolates at New York City hospital laboratories

| No. of isolates | 1993 No. (%) | 1994 No. (%) | 1995 No. (%) |
|---|---|---|---|
| Screened with oxacillin disk[a] | 3,227 | 4,133 | 4,912 |
| zone size ≤ 19 mm[b] | 273 (9) | 549 (13) | 995 (20) |
| Screened and confirmed with approved MIC[c] | 1,229 | 2,491 | 3,535 |
| zone size ≤ 19 mm | 154 (13) | 350 (14) | 704 (20) |
| I[d] | 70 (6) | 209 (8) | 310 (9) |
| R[d] | 19 (2) | 115 (5) | 222 (6) |
| Sterile-site isolates screened and confirmed with MIC[e] | 462 | 649 | 1,321 |
| I | 16 (3) | 66 (10) | 83 (6) |
| R | 5 (1) | 20 (3) | 65 (5) |
| Nonsterile isolates screened and confirmed with MIC[f] | 456 | 662 | 1,596 |
| I | 12 (3) | 83 (13) | 200 (13) |
| R | 5 (1) | 31 (5) | 110 (7) |

[a]Number of laboratories reporting oxacillin disk diffusion test results in 1993, 1994, 1995 was 33, 40, 51, respectively.
[b]Oxacillin disk diffusion test zone size.
[c]Approved penicillin MIC tests include antibiotic gradient strips and broth dilution or micro-dilution using Mueller-Hinton broth supplemented with 2-5% lysed horse blood. Number of laboratories reporting MIC test results in 1993, 1994, 1995 was 10, 22, 35, respectively.
[d]I=Penicillin-intermediate (MIC ≥ 0.1 and ≤ 1.0 µg/ml); R=Penicillin-resistant (MIC ≥ 2.0 µg/ml).
[e]Number of laboratories reporting sterile site isolate results in 1993, 1994, 1995 was 8, 12, 32, respectively.
[f]Number of laboratories reporting nonsterile site isolate results in 1993, 1994, 1995 was 5, 9, 28, respectively.

Table 2. Penicillin susceptibility testing protocols for *Strepococcus pneumoniae* in New York City hospital laboratories

| No. of laboratories[a] | 1993 No. (%) | 1994 No. (%) | 1995 No. (%) |
|---|---|---|---|
| Screened with oxacillin disk | 55 (82) | 63 (97) | 65 (97) |
| Screened all isolates | 38 (57) | 56 (86) | 59 (88) |
| Screened only sterile | 9 (13) | 6 (9) | 6 (9) |
| Other | 8 (12) | 1 (2) | 0 |
| Performed penicillin MIC test | 39 (58) | 51 (78) | 56 (84) |
| MIC-tested | | | |
| All isolates | 10 (15) | 9 (14) | 7 (10) |
| Oxacillin-resistant only | 18 (27) | 39 (60) | 42 (63) |
| Sterile isolates only | 5 (7) | 1 (2) | 0 (0) |
| By request only | 4 (6) | 1 (2) | 5 (7) |
| Other | 2 (3) | 1 (2) | 2 (3) |
| Penicillin MIC technique[b] | | | |
| Antibiotic gradient strip[c] | 11(16) | 41 (63) | 50 (75) |
| Automated tests[c] | 21 (31) | 9 (14) | 2 (3) |
| Broth dilution | 8 (12) | 11 (17) | 5 (7) |
| Unknown | 2 (3) | 3 (5) | 0 |
| Conformed with NCCLS guidelines[d] | 15 (22) | 38 (58) | 46 (69) |

[a]Total number of laboratories responding to survey in 1993, 1994, 1995 was 67, 65, 67, respectively.
[b]13 laboratories used more than one MIC technique.
[c]Antibiotic gradient strip: e.g., E-test. Automated tests: e.g., Microscan, Vitek.
[d]NCCLS=National Committee for Clinical Laboratory Standards. Screened all *S. pneumoniae* isolates with oxacillin disk diffusion test and confirmed resistance with approved penicillin MIC test.

ESC such as cefotaxime or ceftriaxone (ESC-intermediate MIC = 1 µg/ml; ESC-resistant MIC ≥ 2 µg/ml) (6). We will use the term “nonsusceptible” to refer to both intermediate and resistant isolates.

### Findings

The proportion of laboratories conforming with NCCLS guidelines for penicillin susceptibility testing of *S. pneumoniae* increased from 22% in 1993 to 69% in 1995. This was due to an increase in the number of laboratories that screened all isolates, a sharp decrease in the use of automated MIC tests, and a fourfold rise in the use of antibiotic gradient strips for determining MICs (Table 2). Overall, the proportion of isolates with oxacillin disk diffusion test zone size ≤ 19 mm, suggesting possible resistance, increased from 8.5% of isolates in 1993 to 20.2% in 1995 (Table 1). MIC test results showed that 5.7% of isolates were penicillin-intermediate and 1.5% penicillin-resistant in 1993, compared with 8.8% and 6.3%, respectively, in 1995. The prevalence of resistant organisms increased among isolates from both sterile and nonsterile sites and was somewhat higher among nonsterile-site isolates than among sterile-site isolates in 1995 (6.9% vs. 4.9%, Chi-square p = 0.03).

Seven of the laboratories followed NCCLS-recommended methods and provided complete penicillin susceptibility results for both 1993 and 1995. These seven reported that among 779 isolates in 1993, 25 (3.2%) were intermediate and six (0.8%) were resistant to penicillin. Among 896 isolates in 1995, 83 (9.2%) were intermediate, and 47 (5.2%) were resistant.

The prevalence of penicillin resistance varied between laboratories and by geographic area. At

35 laboratories in 1995, 0% to 80% (median 13.1%) of isolates were nonsusceptible to penicillin. Laboratories in the New York City borough of Manhattan reported that 18% of isolates were nonsusceptible to penicillin, compared with 16% in Queens, 14% in the Bronx, and 10% in Brooklyn (MIC data from Staten Island were not available).

In 1995, 18 laboratories reported that of 275 *S. pneumoniae* isolates nonsusceptible to penicillin, 90 (33%) were also nonsusceptible to an ESC.

### Limitations

The survey design had several limitations. First, levels of expertise varied among laboratories regarding antibiotic susceptibility testing of pneumococci. Laboratory variation may partly account for the wide range in the proportion of isolates nonsusceptible to penicillin observed at individual laboratories, which could in turn influence citywide estimates. We did not collect and test isolates to confirm laboratory results. Second, the number of laboratories included in our results increased each year as more laboratories adopted NCCLS methods and provided complete data. This improved the accuracy of the survey but made interpreting trends difficult. Third, citywide estimates inevitably mask important differences in the risk for drug-resistant *S. pneumoniae* infections in specific subpopulations; for example, day-care attendance and prior antibiotic use have been associated with drug-resistant pneumococcal infections in children (1,7). Finally, since information was collected on isolates, rather than individual patients or infections, actual disease incidence could not be calculated.

### Conclusions and Recommendations

Our results document a marked improvement in penicillin susceptibility testing protocols for *S. pneumoniae* during this period. Because of an increase in the proportion of isolates tested and widespread adoption of antibiotic gradient strips for MIC testing, more than two-thirds of laboratories conformed with NCCLS guidelines in 1995, compared with fewer than one-fourth in 1993.

The survey demonstrates that drug-resistant pneumococci are prevalent and may be increasing in New York City. Penicillin resistance increased fourfold between 1993 and 1995, and data from 1996 indicate that it has remained at this level or continued to increase. Nine percent of blood isolates in 1996 were intermediate and 6% resistant to penicillin (New York City Department of Health, unpub. data), compared with 6% and 5%, respectively, among sterile-site isolates in our survey for 1995. The proportion of *S. pneumoniae* isolates resistant to penicillin in New York City was similar to U.S. national averages during this period (2-4). Our finding that 33% of penicillin-nonsusceptible *S. pneumoniae* isolates were resistant to ESCs is also similar to the 29% (4) observed nationally.

On the basis of these results, we recommended that New York City clinicians consider carefully the possibility of penicillin and ESC resistance when treating suspected *S. pneumoniae* infections. Although the response to therapy may vary by route of administration, site of infection, and age and immune status of the patient, treatment failure is increasingly common and can have serious consequences (8,9). Educational efforts are needed to promote appropriate use of antibiotics and encourage use of the 23-valent pneumococcal vaccine in populations at increased risk for pneumococcal disease (10).

This laboratory survey was a relatively low-cost method of estimating the prevalence of drug-resistant pneumococci in New York City. It may serve as a model in areas where clinical laboratories routinely perform drug-susceptibility testing but the resources to collect or test isolates centrally for surveillance are limited. Despite the survey's limitations, the estimates provided may be sufficient to guide clinicians in selecting appropriate empiric therapy for suspected pneumococcal infections. Considerable effort was spent each year disseminating these data to laboratories, hospital infection control departments, infectious disease physicians, pediatricians, and other primary care physicians.

Our ability to expand this survey approach beyond *S. pneumoniae*, penicillin, and ESCs is limited by constraints on hospital laboratory staff. As laboratories computerize and standard formats are developed for the electronic transmission of laboratory test results, collection and dissemination of susceptibility data will be possible on a wider range of pathogens and antimicrobial drugs.

### Acknowledgments

The authors thank Alexandra Dow for her contributions to the 1993 survey and laboratory staff throughout New York City, whose time and effort made this project possible.

**Richard Heffernan, Kelly Henning, Anne Labowitz, Annette Hjelte, and Marcelle Layton**
New York City Department of Health,
New York, New York, USA

### References

1. Hoffman J, Cetron MS, Farley MM, Baugham WS, Facklam RR, Elliott JA, et al. The prevalence of drug-resistant *Streptococcus pneumoniae* in Atlanta. N Engl J Med 1995;333:481-6.
2. Breiman RF, Butler JC, Tenover FC, Elliott JA, Facklam RR. Emergence of drug-resistant pneumococcal infections in the United States. JAMA 1994;271:1831-5.
3. Doern GV, Brueggemann A, Holley HP, Rauch AM. Antimicrobial resistance of *Streptococcus pneumoniae* recovered from outpatients in the United States during the winter months of 1994 to 1995: results of a 30-center national surveillance study. Antimicrob Agents Chemother 1996;40:1208-13.
4. Butler JC, Hoffmann J, Cetron MS, Elliott JA, Facklam RR, Breiman RF, et al. The continued emergence of drug-resistant *Streptococcus pneumoniae* in the United States: an update from the Centers for Disease Control and Prevention's pneumococcal sentinel surveillance system. J Infect Dis 1996:174:986-93.
5. Cetron MS, Jernigan DB, Breiman RF, The DRSP Working Group. Action plan for drug-resistant *Streptococcus pneumoniae*. Emerg Infect Dis 1995;1:64-5.
6. National Committee for Clinical Laboratory Standards. Methods for dilution antimicrobial susceptibility tests for bacteria that grow aerobically. Approved standard. M7-A4. Villanova (PA): The Committee; 1997.
7. Arnold KE, Leggiadro RJ, Breiman RF, Lipman HB, Schwartz B, Appleton MA, et al. Risk factors for carriage of drug-resistant *Streptococcus pneumoniae* among children in Memphis, Tennessee. J Pediatr 1996;128:757-64.
8. Appelbaum PC. Epidemiology and in vitro susceptibility of drug-resistant *Streptococcus pneumoniae*. Pediatr Infect Dis J 1996;15:932-9.
9. Chesney PJ, Wilimas JA, Presbury G, Abbasi S, Leggiadro RJ, Davis Y, et al. Penicillin- and cephalosporin-resistant strains of *Streptococcus pneumoniae* causing sepsis and meningitis in children with sickle cell disease. J Pediatr 1995;127:526-32.
10. Munford RS, Murphy TB. Antimicrobial resistance in *Streptococcus pneumoniae*: can immunization prevent its spread? J Investig Med 1994;42:613-21.
